# Extending the Biopsychosocial Conceptualisation of Chronic Post Surgical Pain in Children and Adolescents: The Family Systems Perspective

**DOI:** 10.1080/24740527.2022.2038032

**Published:** 2022-04-28

**Authors:** Toby Newton-John

**Affiliations:** Discipline of Clinical Psychology, Graduate School of Health, University of Technology, Sydney, Australia

**Keywords:** Family, pediatric pain, surgery, chronic pain, biopsychosocial

## Abstract

A substantial number of children and adolescents undergoing surgical procedures, as many as 40% in some estimates, will go on to develop chronic postsurgical pain (CPSP). Because of the significant negative impact of CPSP on social and emotional milestones, as well as the child’s quality of life, it is important to identify modifiable factors that are associated with the onset and maintenance of the condition. Research has demonstrated that parent factors can play a role in pediatric chronic pain; however, there has been little examination of parent and family influences on the transition to CPSP. Family systems theories, which consider the influence of the family unit overall on the behavior of individuals members, have been applied to the eating disorders literature for decades. This narrative review proposes a novel application of family systems theory to pediatric CPSP and, in particular, highlights the role that parental dyadic factors may play in the development and maintenance of persistent pain following surgery in children and adolescents.

## RÉSUMÉ

Un nombre considérable d'enfants et d'adolescents subissant des interventions chirurgicales, allant jusqu'à 40 % selon certaines estimations, développera une douleur chronique post-chirurgicale (DCPC). En raison des répercussions négatives importantes de la DCPC sur les jalons sociaux et émotionnels, ainsi que sur la qualité de vie de l'enfant, il est important de déterminer les facteurs modifiables qui sont associés à l'apparition et au maintien de la maladie. La recherche a démontré que les facteurs parentaux peuvent jouer un rôle dans la douleur chronique pédiatrique; cependant, l’influence des parents et de la famille sur la transition vers la DCPC a été peu étudiée. Les théories des systémes familiaux, qui étudient l'influence de l'unité familiale dans son ensemble sur le comportement de chacun de ses membres, sont appliquées à la littérature sur les troubles de l'alimentation depuis des décennies. Cette revue narrative propose une application novatrice de la théorie des systémes familiaux à la DCPC pédiatrique. En particulier, elle met en évidence le rôle que les facteurs dyadiques parentaux peuvent jouer dans le développement et le maintien d'une douleur persistante aprés une intervention chirurgicale chez les enfants et les adolescents.

## Introduction

Pain following a surgical procedure is normal and expected, with a relatively linear relationship between the size and scope of the surgery and the amount of discomfort and limitation to function experienced afterward.^1^^,^^2^ However, for a proportion of children and adolescents undergoing surgery, their pain experience and impact on function do not resolve as the body tissues heal. They continue to experience pain for many months and potentially years after their operation took place. Chronic postsurgical pain (CPSP) is defined as pain located in the region of the surgery, where alternative causes such as infection or malignancy have been excluded; pain that persists for three months or longer; and where there are impacts on quality of life.^3,4^ Estimates of the prevalence of CPSP in pediatric populations vary, but in general the 12-month postoperative rates are in the range of 20%^5,6^ to approximately 40%.^7,8^ Considering that each year close to 4 million surgeries are carried out on children aged 0 to 17 years in the United STates alone,^9^ the size of the problem cannot be underestimated.

There are multiple sequelae that result from living with intractable pain for these children, and in many ways these mirror the impacts that chronic pain can have on adults.^10,11^ Increased psychological distress, difficulties carrying out activities of daily living, impaired sleep, frequent health care attendances, and increased medication consumption are all commonly associated with pediatric CPSP.^5,7,12–14^ However, there are additional complexities for children and adolescents experiencing persistent pain that relates to their developmental phase. School attendance is often impacted by chronic pain, and interruptions to regular schooling can have major consequences beyond the immediate academic disruption.^14–16^ For example, Eccleston and colleagues sampled 110 adolescents with chronic pain and discovered that as a group they perceived themselves to be significantly less developed than their peers on a range of adolescent social development factors, including their sense of independence, emotional adjustment, and identity formation. Results such as these reinforce the importance of addressing the transition from acute to chronic pain in pediatrics, because the consequences can extend across multiple social and emotional developmental domains.

Research investigating CPSP has identified a number of biological, social, and psychological variables that are predictive of the transition from acute to chronic pain. The adult literature has shown that older age, female gender, and presurgical pain intensity are reliably related to the development of CPSP.^17,18^ However, in a systemic review of pediatric CPSP by Rabbitts et al., the only reliable biological predictor of persistent postoperative pain was presurgical pain levels.^5^ In a later comprehensive predictive study by Rosenbloom and colleagues,^7^ the only variable that predicted both pain intensity and pain unpleasantness at 12 months was baseline functional disability, suggesting that different factors may be relevant when considering the transition to chronicity for children compared to adults.

An intriguing line of research exploring factors predictive of ongoing pain in children many months after surgery concerns the child’s memory for pain, specifically the accuracy of their recall. In this approach, children undergoing surgical procedures are asked to complete pain intensity scores for three consecutive days postoperatively (which are averaged), and they are then contacted at a later period and asked to recall how much pain they remember having been in after their procedure. More negatively biased recall of pain, where recalled pain is higher than the initial pain report, has been shown to predict higher reports of pain 2 months postoperatively^19^ which is the period of concern for developing CPSP. Unpacking this phenomenon further, Noel and colleagues^20^ demonstrated that greater anxiety sensitivity at baseline, which, according to cognitive processing theory should result in selective attention to, and greater encoding of, threatening information leading to an overestimation in pain recall, was indeed related to greater negatively biased recall of pain. Further, the greater the negatively biased recall, the higher the pain intensity at 6 and 12 months postsurgery.

Studies such as these are shedding new light on the intrapersonal predictors of CPSP; however, there is preliminary evidence that parents might also play a role in these cognitive bias processes. For example, it has been shown that greater parental use of pain words when reminiscing about their child’s experience of tonsillectomy is associated with more negatively biased pain memories held by the child,^21^ underscoring the importance of the family context when considering the long term conditions such as CPSP.

It is also important to point out, as Katz and Selzer have noted,^18^ that there is a difference between processes that promote the *development* of chronic pain following an acute episode and processes involved in the *maintenance* of chronicity for extended periods after the onset surgical event. Most of the research in the pediatric pain arena has investigated maintaining factors (e.g., Donnelly et al.^22^) rather than those potentially involved as originators of chronic pain development.

In addition to predictors of CPSP that relate to the child themselves, research has begun to explore interpersonal influences on CPSP development, in particular parental factors. One of the areas that has received the most research attention is that of parental overprotectiveness. Though exhibiting a degree of caution and concern toward one’s child is adaptive and appropriate, especially in circumstances of physical challenge such as after an injury or in the context of a disease, parental caring behaviors can become excessive and prolonged and ultimately create harm. Among children with chronic pain, parental overprotectiveness has a similar effect to spouse solicitousness in adult dyads, in that it is reliably associated with greater functional disability^23,24^ as the parent attempts to prevent the child from engaging in behaviors that he or she considers risky or harmful but that engender lower self-efficacy and increased physical dependence in the long term. Wilson and Fales^25^ identified factors such as parental guilt, reduced involvement in the child’s activities, and parental inconsistency as the drivers of protective behaviors in chronic pediatric pain. Though parental overprotectiveness has not yet been examined in relation to CPSP,^19^ this article will highlight how various factors suggest it is likely to be relevant.

Parent trait anxiety level has also been shown to be a predictor of negative child pain outcomes after surgery,^26^ and parent presurgical anxiety sensitivity was predictive of child functional disability levels 12 months postsurgery.^27^ In a longitudinal study of 76 children undergoing corrective surgery for scoliosis, Siemer et al.^28^ found that 20% of the variance in the child’s 12-month pain interference score could be accounted for by parent factors, notably pain catastrophizing and parental preference for using analgesia (i.e., the preference to relieve pain rather than avoid the risks associated with analgesic use). However, in an interesting application of the actor–partner interdependence modeling technique, Birnie and colleagues^29^ found that for a sample of youth undergoing spinal fusion surgery, the child’s own catastrophizing score predicted his or her pain levels pre- and postsurgically but the parent’s catastrophizing score did not. This may have been that because it was an adolescent sample (mean age 14.5 years) and parental cognitive influences were less pronounced than with younger children. Nevertheless, these data point to the relevance and importance of parent factors in the development of pediatric CPSP. However, research in this area has consistently suffered from a number of limitations relating to the design and theoretical underpinnings of the studies themselves.

Firstly, almost all of these studies have been conducted with the mother as the parent and, as a result, the influence of the father on pediatric CPSP remains largely unknown. There is some evidence that fathers’ interactions with children with chronic pain are different than those of mothers^23,30^ and, as such, this is an important omission in the literature. It is also the case that much of this literature has been based on cross-sectional, correlational analyses of child cognition/affect/behavior and parent cognition/affect behavior and the influence of the parent on the child is inferred from these associations. These unidirectional analyses are a simplification of the much more complex, *reciprocal* interactions that occur within close relationships such as a parent and child. As has been noted by Rabbitts and Fisher,^31^ “Dyadic interactions between parent and child are considered key in the maintenance of pain” (p. 1848), yet few studies employ research methodologies such as the actor–partner interdependence model^32,33^ that take into account this two-way, dyadic influence.

Finally, and related, the pediatric CPSP literature has been limited by its relative ignorance of the influence of the parental dyad and wider family environment on children’s postsurgical recovery.^4^ Unlike in the adult literature, where interpersonal factors such as social and relationship support are routinely considered as influential factors in the maintenance of chronic pain^34,35^ and have been considered as predictors of adult CPSP,^17,36^ the pediatric literature has not explored the quality of the parental relationship and other family interaction variables as possible influences on CPSP.

This is not to suggest that these broader family constructs have not been considered at all in terms of child health outcomes. The pediatric eating disorders literature has been pursuing a family-based assessment and treatment model for more than 50 years,^37^ and the remainder of this review will consider the application of this literature to the issues of CPSP and whether there are useful lessons to be learned.

## Family Systems Models

Family systems theories, of which there are a number, have in common the central premise that families function as a unit, rather than a series of individuals interacting independently of each other. The behavior of each member of the unit inevitably influences the behavior of every other member in the unit.^38–40^ This is the so-called principle of nonsummativity^41^—there is no straightforward sum of the parts within family systems theories but a “third” reality involving a child’s behavior, the parent’s behavior, and their relationship.^41^ Within the systemic perspective, the object of study moves from a focus on the individual, or from the dyadic mother–child relationship, to the entire system of interactions in which the members of the family nucleus live.

One of the earliest family systems models to be applied to the problems of pediatric illness was Minuchin’s “psychosomatic family” model.^37,42^ In this model, families with certain key characteristics—*enmeshment* (inadequate boundaries between family members), *overprotectiveness* (excessive concern for each other’s welfare), *rigidity* (limited adaptability within the family to changing circumstances), and *lack of conflict resolution* (an absence of negotiation skills within the family, including conflict avoidance behaviors)—were said to be overly represented in children with long-term illness. The theory was initially applied to children living with poorly controlled type 1 diabetes and later to children who developed anorexia nervosa. Subsequently, the McMaster model^43,44^ identified six generally applicable areas of family functioning: problem solving, communication, roles, affective responsiveness, affective involvement, and behavioral control. Families are said to operate on a continuum from effective to ineffective on each of these six dimensions.^44,45^ Finally, the process model of family functioning^40^ also defines six universal family criteria, but the emphasis here is upon the interactions between the family functioning domains rather than the structure of the domains themselves. Domains include *task accomplishment, role performance*, and *values and norms*.

Importantly, family systems theories also generally embrace the notion of “homeostasis,”^39,41,45^ whereby the family unit is driven to maintain balance and consistency. This can result in a resistance to changing patterns of interaction, even when they are dysfunctional, because of the desire to maintain family structures that are familiar and predictable. According to some family systems theorists, Kazak^46^ a child’s symptoms in the context of a poorly functioning family can be reframed as “solutions gone awry”; in other words, the child’s symptoms are a form of solution for a family who otherwise is unable to resolve conflict or develop trusting relationships with each other or the health care staff they interact with.

## Family Systems and Eating Disorders

The later stages of childhood and adolescence involve developmental phases in which important social, behavioral, and emotional–motivational changes occur.^41,47,48^ Because of the significance of these changes, the eating disorders literature has long identified that this is an at-risk period for the onset of mental health difficulties,^49,50^ and the same could be said for challenges in terms of managing physical health difficulties, including chronic postsurgical pain.

Family systems models initially conceptualized negative relationships between family members as critical in the genesis of eating disorders. In particular, families of children who developed anorexia nervosa were thought to display excessive levels of enmeshment and rigidity^50^ and that it was the adolescent’s normal developmental need for autonomy being thwarted by the pathological family system that produced his or her symptoms.

The original “psychosomatic family” formulations were based on clinical observation rather than empirical methods, and evidence has since determined that there are no distinct forms of family behavior that relate to the development of eating disorders, irrespective of the diagnostic type.^45,47,51^ Moreover, family systems models have been accused of parent blaming and adding to the distress and angst that already exist in families where a child has significant health concerns.^41,52^ The pathogenesis of eating disorders is now recognized as being multifactorial and includes a variety of genetic, psychological, neuroendocrine, social–cultural and family factors.^53^

Researchers have also examined whether dysfunctional family systems may be relevant in the maintenance of child’s disordered eating, once the behavior has been established.^45^ There is evidence that excessive dependence on other family members, poor communication, and avoidance of conflict are associated with unhealthy weight-related behaviors, especially among daughters.^50,54,55^ However, these family characteristics may be as much a consequence of living with significant illness as maintain it and thus causality cannot be inferred.

Nevertheless, contemporary approaches to the treatment of eating disorders in children and adolescents incorporate the family as part of a comprehensive treatment plan. In fact, parental involvement has been described as a sine qua non of child and adolescent eating disorders treatment.^47^ The Canadian Practice Guidelines panel made a “strong recommendation” for the provision of family-based treatment in eating disorder interventions,^56^ and Jewell has stated that family therapy is “firmly established” as an adjunctive eating disorders intervention.^52^ Broadening the focus of treatment to include the wider family context is not unique to children and adolescents with eating disorders, however. It is also now recommended for the treatment of a number of pediatric conditions, including anxiety disorders^57^ and obesity management^58^; however, it is some way from being standard practice in the treatment of pediatric persistent pain.

## The Application of Family Systems Theory to Pediatric Chronic Postsurgical Pain

As noted, normal adolescent development is characterized by a progressive independence from parents and other family caregivers. Yet because most adolescents still live with their parents and are financially dependent on them, there is an inherent strain. Furthermore, for an adolescent who has undergone a major pain-precipitating event such as surgery, there will naturally be greater dependence on caregivers during the recovery phase. This could potentially place even greater strain on the need for increased autonomy that characterizes this developmental phase.^48,59^ All of these factors underscore the importance of parent and family functioning in any consideration of pediatric emotional and or physical health.

### Chronic Pain Families and CPSP

Taking a family systems analysis approach to pediatric CPSP involves firstly considering those factors that are unique to families in this situation. For example, given that approximately 20% of the adult population report chronic pain,^60,61^ the probability of at least one parent of child with an acute pain issue also having a chronic pain condition is relatively high. The salience of this is that a systematic review and meta-analysis by Higgins et al.^62^ showed that there was a greater prevalence of chronic pain in children where either or both parents had chronic pain. A recent longitudinal study of 11,863 children by Voepel-Lewis and colleagues^63^ also found that parent symptom burden (which includes the presence of pain) was a significant predictor of child persistent pain at 12 months.

Though there may be multiple genetic and/or early neurobiological development factors at play here,^63,64^ it is also true that a variety of family environment factors may also be contributing to chronic pain chronicity. Family systems theorists in chronic pain^20^ have described a chronic pain version of the “family homeostasis” concept previously referred to. For example, because chronic pain often involves a reduction or withdrawal from social interaction and community involvement, the family benefits from having one member incapacitated due to pain, which allows the family to “bind together” and manage the social isolation that would otherwise be a source of regret and frustration. Family systems theorists also refer to the maintaining effect of chronic pain on the sense of identity for members within the family, such that individuals who otherwise have not developed a separate identity from their family come to be seen and treated as the person with pain.^65,66^ It should be noted, however, that there is also research to suggest that youth with chronic pain are very aware of, and struggle against, the influence that pain has on their identity development.^67^

Parents with chronic pain may be overly protective of their child during postsurgical recovery; for example, urging extreme caution with their child’s physical therapy because of their own negative pain experiences with that treatment approach. As previously noted, parental overprotectiveness is associated with greater functional disability in children with established chronic pain,^23,24^ and parents living with pain are more protective in relation to their children’s pain than those without,^25^ so the potential for influence in CPSP development is clear. Alternatively, given the high prevalence of depressive illness in those with chronic pain,^68^ children of patients with chronic pain may not receive adequate postoperative support due to lack of parental availability, as noted by Wilson and Fales,^25^ thereby increasing the risk of CPSP development.

The way in which childhood attachment bonds are formed is known to have a profound influence on later psychosocial functioning, and parent–child attachment styles have also been considered in the transition from acute to chronic pain in children.^69,70^ It has been argued that because a surgical procedure in effect represents an acute physical threat, it may activate attachment-based behaviors and hence escalate potential vulnerabilities with the parent–child dyad.^65^ Adding to the complexity here is that the attachment vulnerabilities may reside in the parent, the child, or both. For example, Kerley et al.^70^ recently suggested that avoidantly attached children may be perceived by their parents as not wanting or needing support and hence receive relatively low levels of protective parenting. In the present scenario, this could become problematic if the child does not cope with the postoperative pain and distress. Equally, the experience of their child undergoing major surgery may activate overly protective behaviors in anxiously attached parents, and this could generate the kinds of solicitous responses discussed previously that are associated with dependence and disability in both children and adults with chronic pain.^24,71^ The effect of attachment styles of parents and children on CPSP development have yet to be empirically examined; however, the evidence suggests this would be a worthwhile avenue to pursue.

Social modeling can also take place in chronic pain families.^64^ From a family systems perspective, this would be conceptualized more broadly than the child just observing operant behavioral reinforcement taking place.^71–73^ As was previously mentioned, people living with chronic pain whose partners are highly solicitous (i.e., they respond to pain behaviors in positively reinforcing ways, such as giving a massage or fetching pain medications, or in negatively reinforcing ways, such as releasing the individual from unwanted activities like domestic chores) are also more functionally disabled by pain.^34,71^ In a chronic pain family characterized by high solicitous/high disability marital dyad, the child or adolescent with postsurgical pain may watch their parents’ interactions and learn that the expression of pain behaviors leads to desirable responses and hence adopt them as well.

More recent work has further suggested that solicitous responses can function as more than just an appetitive or avoidance reinforcer. Cano and Williams^74^ cogently argued that verbal expressions of pain can be conceptualized as self-disclosures that, when met with a solicitous response, function to build intimacy within the relationship. Though the notion of pain-related interactions operating to enhance relationship functioning has only been examined in adult relationships thus far,^75,76^ it is not difficult to envisage a similar process occurring in the context of pediatric CPSP.

For example, take the common situation where an adolescent whose need for autonomy was causing friction within the family system (perhaps because the parents were inflexible, had limited affective responsiveness, and had poor conflict resolution skills). The disharmony between the adolescent and his or her parents was causing stress to the other children in the family, which in turn created further guilt in the parents and additional stress for the adolescent. However, following the adolescent undergoing surgery, which limited their mobility for a period of time, the parents’ solicitous behavior toward their child provided practical support *but also emotional validation*, perhaps for the first time in many months. The arguments cease, the parents regain control, the impact on the other children in the family is removed, and the adolescent’s needs for an emotional bond with the parents are met at a time when the pursuit of autonomy is curtailed by the need for convalescence. Hence, homeostasis within the family unit is restored, as long as the adolescent requires the parents to continue providing that practical support. So the expressions of pain behavior are reinforced and the adolescent’s pain-related disability steadily increases.

### General Family Factors

Moving on from pain-specific family factors, a family systems approach to pediatric CPSP would also take into account other patterns of interaction within the family unit. A step that has already been taken in this direction is the interpersonal fear avoidance model developed by Simons and colleagues,^23^ which demonstrated that parent fear of pain and overly protective behavior significantly contribute to child functional disability levels. The study also showed that these effects were bidirectional, such that the child’s catastrophizing also influenced the parent’s pain-related fears. Though results such as these are aligned with a family systems perspective, the study sample involved 91% children with established chronic pain (and 92% mothers and 75% daughters), so its applicability to general CPSP development is not clear.

Few studies have directly tested the influence of family factors on pediatric CPSP, and the results thus far have been mixed.^4^ Whereas it has been found that parental pain beliefs can influence the onset of CPSP in some cases,^77,78^ a number of studies have not shown parent factors to be relevant to CPSP.^4,7,8^ However, given the strong theoretical frameworks advanced by various family systems models and the evidence supporting the role that parental factors play in the maintenance of chronic pediatric pain,^79^ it is reasonable to consider whether systemic family factors can influence pediatric CPSP. As noted by Simons and colleagues,^23^ “At the broadest level, … findings underscore parents as a key context for understanding, assessing, and managing pediatric pain, and provide evidence for the bidirectional relationship between parent factors and child functioning” (p. 702).

Having identified that there a range of family factors that may influence the development of pediatric CPSP, the issue becomes whether interventions in order to prevent or limit the onset of the condition are possible. At one end of the scale, a service such as the Toronto General Hospital Transitional Pain Service^80^ offers comprehensive multidisciplinary input to target the biopsychosocial factors that are relevant to CPSP development. The service did not initially include children and adolescents as patients; however, there are plans for their integration.^80^ However, it may not require a tertiary referral specialist service in order to provide effective interventions for children and adolescents at risk of developing CPSP. The pediatric chronic pain literature shows that including parents in treatment produces better outcomes,^81^ and there is no reason that family-based interventions for young people at risk of CPSP should not also be effective. Drawing upon family systems models, such interventions would likely target the kinds of factors that have been discussed, in particular, parental overprotectiveness and pain anxiety, family conflict resolution skills and communication, and the flexibility and adaptability of the family unit toward change.^42^ Intervention modalities would include cognitive behavioral^79^ as well as systemic family therapy approaches^65^ and may additionally incorporate attachment-based therapeutic techniques.^82^

## Future Research

One of the limitations of the current pediatric pain literature is that though several family functioning assessment tools exist, including the Family Environment Scale,^83^ The Family Adaptability and Cohesion Scale,^84^ and the McMaster Family Assessment Device,^85^ their utility for assessing CPSP outcome is limited. They are lengthy to administer (for example, the McMaster scale is 60 items), they lack normative data against which to compare results from clinical samples, and psychometric support regarding predictive validity and responsiveness to change is scant.^86^

There are a number of other questions that future family systems research as applied to pediatric CPSP should consider. The parental dyad, and particularly the state of the primary caregivers’ relationship, is critical. The adult chronic pain literature has shown that marital satisfaction often moderates the effects of pain on quality of life,^87^ and it may also be the case that parental influences on CPSP are moderated by relationship harmony between the caregivers. As an example, family systems theory recognizes the concept of “scapegoating” in dysfunctional families, whereby the parents can avoid addressing their own interpersonal difficulties by directing all of their attention toward one member of the family unit.^88^ It is conceivable that a similar excessive focus on a child recovering from surgery as a diversion from parental relationship difficulties could function to generate or maintain CPSP in the child. This is an example of the family systems model applying to younger children undergoing major surgery, whereas previous examples of CPSP and family homeostasis have related more to adolescent postoperative care.

As has already been stated, the majority of studies in the pediatric pain literature have been conducted with mothers as the respondent.^30,33,89^ To what extent fathers influence the family system in relation to CPSP remains to be seen. It is also not known how family systems respond to different kinds of surgical events and what impact that has upon later recovery from the operation. For instance, does the family unit respond differently when the child undergoes surgery for a sudden, life-threatening condition, such as resection of a malignant tumor, compared to the correction of a benign condition that was anticipated and planned for, such as a scoliosis? Future investigations might explore whether differential responses to these kinds of surgical situations have an impact on CPSP outcomes.

And finally, pediatric pain research has only recently begun to explore the role of the sibling in childhood adjustment to pain.^90,91^ Much of the work to date has focused on the genetic influence or vulnerability of the sibling, rather than exploring sibling influences and experiences from a family systems perspective. Of particular relevance to the present discussion is the degree to which the CPSP trajectory is influenced by the presence of a sibling(s). It is possible that in families where there are other children also requiring parental attention there is a dilution of parental reinforcement of pain-related disability and hence the presence of siblings may be “protective” against CPSP. It is equally possible that a child might model pain behavior from his or her siblings (and not just parents as was noted previously), and this might increase the probability of developing CPSP. The extent to which siblings can provide support for a young family member living with chronic pain is also a question of interest for future researchers to explore.

## Conclusions

Given the potentially devastating consequences that CPSP can have on pediatric development and quality of life, it is critically import that research continues to work toward identifying those modifiable factors that are associated with its onset and maintenance. Family systems models offer a theoretically driven perspective by which to interpret the complex, reciprocal interactions that occur within every family. There is a long tradition in the eating disorders literature of considering child and adolescent clinical presentations from the perspective of the family, rather than just the individual, and the evidence supports the adoption of this more holistic view. Though care must be taken not to attribute blame to the family when formulating the factors relevant to problem development and maintenance, eating disorder interventions in this population now routinely include the broader family perspective. Assessment will necessarily be more extensive when undertaken from a family systems approach; however, the potential benefits in terms of detecting and then intervening to prevent CPSP are undeniably worthwhile.
